# Competition-cooperation mechanism between *Escherichia coli* and *Staphylococcus aureus* based on systems mapping

**DOI:** 10.3389/fmicb.2023.1192574

**Published:** 2023-11-06

**Authors:** Caifeng Li, Lixin Yin, Xiaoqing He, Yi Jin, Xuli Zhu, Rongling Wu

**Affiliations:** ^1^Center for Computational Biology, College of Biological Sciences and Technology, Beijing Forestry University, Beijing, China; ^2^National Engineering Laboratory for Tree Breeding, Beijing Forestry University, Beijing, China; ^3^Key Laboratory of Genetics and Breeding in Forest Trees and Ornamental Plants, Ministry of Education, Beijing Forestry University, Beijing, China; ^4^The Tree and Ornamental Plant Breeding and Biotechnology, Laboratory of National Forestry and Grassland Administration, Beijing Forestry University, Beijing, China

**Keywords:** *Escherichia coli*, *Staphylococcus aureus*, systems mapping, competition-cooperation mechanism, interspecific interaction

## Abstract

**Introduction:**

Interspecies interactions are a crucial driving force of species evolution. The genes of each coexisting species play a pivotal role in shaping the structure and function within the community, but how to identify them at the genome-wide level has always been challenging.

**Methods:**

In this study, we embed the Lotka-Volterra ordinary differential equations in the theory of community ecology into the systems mapping model, so that this model can not only describe how the quantitative trait loci (QTL) of a species directly affects its own phenotype, but also describe the QTL of the species how to indirectly affect the phenotype of its interacting species, and how QTL from different species affects community behavior through epistatic interactions.

**Results:**

By designing and implementing a co-culture experiment for 100 pairs of *Escherichia coli* (*E. coli*) and *Staphylococcus aureus* (*S. aureus*), we mapped 244 significant QTL combinations in the interaction process of the two bacteria using this model, including 69 QTLs from *E. coli* and 59 QTLs from *S. aureus*, respectively. Through gene annotation, we obtained 57 genes in *E. coli*, among which the genes with higher frequency were *ypdC*, *nrfC*, *yphH*, *acrE*, *dcuS*, *rpnE*, and *ptsA*, while we obtained 43 genes in *S. aureus*, among which the genes with higher frequency were *ebh*, *SAOUHSC_00172*, *capF*, *gdpP*, *orfX*, *bsaA*, and *phnE1.*

**Discussion:**

By dividing the overall growth into independent growth and interactive growth, we could estimate how QTLs modulate interspecific competition and cooperation. Based on the quantitative genetic model, we can obtain the direct genetic effect, indirect genetic effect, and genome-genome epistatic effect related to interspecific interaction genes, and then further mine the hub genes in the QTL networks, which will be particularly useful for inferring and predicting the genetic mechanisms of community dynamics and evolution. Systems mapping can provide a tool for studying the mechanism of competition and cooperation among bacteria in co-culture, and this framework can lay the foundation for a more comprehensive and systematic study of species interactions.

## 1. Introduction

In nature, the survival and development of any species are inseparable from its surrounding living environment. The phenomenon of interspecies interactions generally exists between different individuals of the same species, population, or family, and runs through all stages of biological growth and development (Gallagher et al., 1983; Tilman, 1994; Holt and Polis, 1997). A species needs to adapt not only to the physical environment, but also to another species with which it interacts (Wade, 2007). The interspecies interactions can not only affect community structure, organization, and function, as well as adaptation to changing environments (Suttle et al., 2007; Alexander et al., 2015), but also act as an evolutionary force that drives species to change in time and space (De Mazancourt et al., 2008; Whitham et al., 2008; Turcotte et al., 2012). The genes of each coexisting species play a critical role in shaping the internal workings of communities (Whitham et al., 2006; Bailey et al., 2009; Hersch-Green et al., 2011), however, how to identify these genes at the genome-wide level has always been an unsolved problem. While there have been a lot of simple experimental designs which can be able to describe how individual genes or pathways contribute to ecological interactions in communities (Schwarzenberger et al., 2009; Miyakawa et al., 2010; Miner et al., 2012), it is difficult to map a comprehensive genetic architecture of how different species interact and communicate. Despite the increasing availability of genetic data through high-throughput genotyping and sequencing technologies, many studies have used association analysis to locate quantitative trait loci (QTLs) that can influence phenotypes, but traditional genetic mapping focuses on phenotypic variation in an individual species and fails to describe how QTLs determine the formation of multiple species into a community. Meanwhile, the mapping results will also cause problems such as loss of heritability (Mackay, 2014; Uricchio, 2020), and it is still impossible to construct an accurate genotype–phenotype map for interspecific interactions in populations, communities, or ecosystems (Davey et al., 2011).

Microbes have extremely high richness and diversity in ecosystems. They do not exist alone, but coexist with many species in microbial communities and form complex interaction networks (Bell et al., 2005; Falkowski et al., 2008). Such interaction is characterized by diversity and dynamics, which is of great significance in colony formation and response to changes in the external environment, and plays a pivotal role in maintaining the stability of the ecosystem (Faust and Raes, 2012). Many studies have shown that when the environment changes, the interaction between species will affect the community structure, resulting in changes in species abundance, and these changes will react to the ecosystem function in return (Brown et al., 2001). A study has observed that experimental communities of five bacterial species in polyculture were more productive and evolved more rapidly than the same species in monocultures in a novel environment in the laboratory (Lawrence et al., 2012). In the process of interaction, microorganisms secrete some enzymes, growth factors or transmit some signals in order to maintain their own life activities, thereby changing their behavior (Little et al., 2008). Previous studies related to species interactions mostly focused on resource acquisition and utilization, individual physiological responses, and community composition changes during the interaction process. However, in this process, which genes hinder or promote the adaptation of microbes to new environmental conditions, and how to quantify the genetic effects in the interaction process are still issues to be solved.

Complex traits are genetic traits regulated by multiple genes and the environment. Its formation is not only directly affected by its own genes, but also indirectly affected by the genes of other individuals in the population, as well as affected by the epistasis interactions between genes between different individuals (Whitham et al., 2008; Lambrechts, 2010). Traditional QTL mapping is a reductionist approach, which can only identify the direct genetic effects of a species’ QTLs on its own phenotype, but cannot detect the indirect genetic effects of a species’ QTLs on the phenotype of interacting species in the same community and genome-genome epistasis effects of different species’ QTLs on community phenotype (Jiang et al., 2018a,b). This type of epistasis can not only occur among QTLs in the same genome, but also among QTLs from different genomes. In molecular genetic experiments, some studies have located such epistatic QTLs dependent on other genomes (Lambrechts, 2010) and also identified the molecular pathways related to the QTL function (Biscarini et al., 2010). Considering a complex trait as a system composed of interactive components, the traditional mapping models can be upgraded to a new approach, named the systems mapping model (Wu et al., 2011; Sun and Wu, 2015). By integrating a set of ordinary differential equations (ODEs), the systems mapping model can not only study dynamic phenotypic data, but also decompose a complex trait into different components (Wu et al., 2011), and then analyze and quantify the dynamic changes of a component and its interaction relationship with other components in a complex system, thus better understanding the underlying mechanisms of trait formation and development. The epistatic interaction between genomes from different individuals accounts for a large proportion of the total genetic variance (Mackay, 2014; Uricchio, 2020), and our model also provides a new perspective to solve the problem of complex trait loss heritability.

In this study, we selected *E. coli* and *S. aureus* as research objects, which are closely related to human health and widely distributed in nature. Dynamic quantitative phenotypic data of two kinds of bacteria in co-culture at 14-time points were detected by quantitative real-time PCR (qPCR), and high-density genetic markers of two bacteria, namely single nucleotide polymorphisms (SNPs), were obtained by whole-genome resequencing, respectively. By correlating dynamic phenotypic data with SNPs data through the systems mapping model, significant QTLs that play a vital role in the interaction between the two bacteria were mapped and the functional annotations of these QTLs were performed. Incorporating the Lotka-Volterra interspecies competition model in the theory of community ecology into the systems mapping, the overall growth of the two bacteria can be decomposed into independent growth and interactive growth, which could be combined with the strategy matrix of game theory to study the mechanism of competition and cooperation among bacteria. The quantitative genetic models were used to verify whether significant QTLs exerted direct effects, indirect effects, and genome-genome epistasis effects between bacterial interactions, and then three kinds of genetic effect networks among key QTLs from two bacteria were constructed to explore the regulatory mode on bacterial interactions. The systems mapping model can provide a new idea and method for studying the QTLs of interactions between bacteria in co-culture, and also provide a theoretical basis for the study of more complex interaction models. This statistical framework can provide a powerful tool for studying the genetic mechanism of complex traits in animals, microbes, and humans, as well as a theoretical basis for a systematic and comprehensive understanding of the nature of bacterial interactions, and also provide new insights for explaining the influence of genetic factors on complex traits.

## 2. Materials and methods

### 2.1. Microbial interaction ecological experiment

In order to study the competition and cooperation mechanism of two species in the same environment, we, respectively, collected 100 strains of *E. coli* and *S. aureus* and randomly paired these strains to form a total of 100 independent interspecific combinations and numbered them. The source information of 200 strains can be found in Supplementary Table S1 and the relevant sequencing information has been uploaded to the National Genomics Data Center (NGDC) database. Two hundred original strains were cultured on the tryptose soya agar (TSA) solid medium (OXOID, Basingstoke, England) for activation treatment. A total of 100 conical flasks (50 mL) were, respectively, added to 25 mL of the same two-times dilution of brain-heart infusion (BHI) medium (OXOID, Basingstoke, England). We picked out the single colonies of two bacteria on the TSA culture plate and then inoculated them to 100 BHI culture media for co-culture according to the numbered pairing principle. The absorbance of the bacterial solution was measured with an enzyme-labeled instrument and converted to the bacterial solution concentration, and the bacterial solution was diluted to 5 × 10^3^ cells/mL, and the flask was cultured continuously for 36 h in a constant temperature shaker at 30°C and 130 r/min. This co-culture experiment was replicated at least three times. The method of randomized block experiment was adopted to eliminate the random error which may be produced by the environment. Faced with the pressure of limited resources, two strains from different species in a culture flask may choose to compete or cooperate.

According to the law of bacterial growth, the strain will show a trend logarithmic growth before the stable period, 1 mL of samples were taken every 0.5 h, 2 h, 4 h, and 6 h at 0–2 h, 2–12 h, 12–24 h, and 24–36 h, respectively, and then bacterial genomic DNA was extracted by using the TIANamp Bacterial Genome Extraction Kit. The specific primers were designed according to the *uidA* (1694260–1,696,071) gene encoding β-D-glucosidase in *E. coli* and the *nuc* (1397756–1,398,289) gene encoding thermostable nuclease in *S. aureus*. The specific fragments obtained by primer amplification were cloned into the pMD18-T vector for preparation of standards, and then these standards were further diluted by 10 times gradient to obtain standard curves. Through the Mx3005P real-time fluorescence quantitative system (Stratagene, La Jolla, United States), two specific primers were used to perform qPCR amplification on *E. coli* and *S. aureus* to obtain CT values. These values were then substituted into the standard curve to calculate the bacterial abundance of each bacteria in each flask at each time point, which was repeated three times to take the average value for genetic mapping. The total volume of the qPCR reaction system was 25 μL, including 2 × SuperReal PreMix Plus (SYBR Green I) (TIANGEN, Beijing, China), 300 nM forward primer, and 300 nM reverse primer. The thermal cycle reaction conditions were as follows: initial denaturation at 95°C for 10 min, followed by 40 cycles of 30 s at 95°C, 1 min at 55°C, and 1 min at 72°C. Fluorescent signals were detected and collected during annealing and extension.

### 2.2. Whole genome sequencing

Using *E. coli* str. K-12 substr. MG1655 and *S. aureus* subsp. aureus NCTC 8325 as reference strains, the whole genome sequencing of 100 strain combinations was performed on the Illumina HiSeq 2,500 (Novogene, Beijing, China) platform to obtain SNPs (single-nucleotide polymorphisms) genotype data of two species at the genome-wide level. The obtained effective sequencing data was performed for the sequence alignment with the reference genome using BWA (Li et al., 2009) software (version 0.7.17) and for quality control to ensure the SNPs data with high-quality scores (Q value ≥20) and enough supporting bases (≥ 4, with variation) using SAMtools (Li et al., 2009; Danecek et al., 2021) software (version 1.17). Ultimately, a total of 745,528,965 SNP combinations were obtained from the *E. coli* and *S. aureus* genomes, which should be sufficient enough in density to identify genomic regions in the QTL mapping.

To obtain high-quality SNP combinations and improve systems mapping efficiency, this study adopted a two-step approach to filter out low-quality SNP combinations: (1) Before combining the SNPs of two bacteria, the single marker analysis method was used to control the number of SNPs, and T-test was used to filter out SNPs below the threshold; (2) After pairing the screened SNPs, SNP combinations were further filtered by comparing the proportions of the four genotypes in each combination. SNP combinations with genotype proportions below 10% were filtered out. Based on the above method, 272,873 high-quality SNP combinations were ultimately obtained, greatly improving the efficiency of parameter estimation for systems mapping.

### 2.3. Analysis of population structure and kinship relationship

Population genetic structure analysis can provide information on the origin and composition of individuals and is a worthwhile tool for analyzing genetic relationships. Based on the posterior variational Bayes framework, fastStructure software (Raj et al., 2014) is able to calculate the estimated values faster than the classical population structure analysis software STRUCTURE, which is suitable for inferring the population structure from large SNP genotype data. In this study, we conducted population structure analysis on SNPs data of two species using fastStructure software (version 1.0). Based on the default convergence criteria and prior probability, K values ranging from 2 to 20 were tested with 10 replicates per K. Using the chooseK.py function in fastStructure, a reasonable range of K values was determined. Meanwhile, the prcomp function in the R software was performed for principal component analysis (PCA) to further investigate the structural components of the two bacteria and their relationship. The emma.kinship function in the R package emma was used to calculate the kinship matrix of two bacterial SNPs used in subsequent analysis (Kang et al., 2008). According to the analysis of kinship and population structure, we used the lmer function in the R package lme4 (Bates et al., 2015) to adjust the phenotype of two bacteria based on the linear mixed effect model, to avoid detecting the phenotypic data of the falsely associated population structure.

### 2.4. Systems mapping model

Assume that there are two QTL that affects the growth trait of two bacteria in co-culture, including two genotypes A and a by species E and two genotypes B and b by species S, respectively. These two QTLs formed four interspecific genotype combinations, expressed as A/B , A/b , a/B , and a/b . By correlating SNPs genotype data and dynamic phenotypic data of n interspecific strains combinations, we used maximum likelihood estimation (MLE) to construct a dynamic model for parameter estimation of specific genotypes. A mixture-based likelihood model has been widely used to map QTLs for complex traits. The mixture-based likelihood of time-dependent abundance data for n interspecific strains combinations is formulated as


(1) $$\begin{array}{l} L\left(N^{E}{\mathrm{,N}}^{S}\right)=\\ \overset{n}{\underset{i=1}{\prod }}\left[\begin{array}{c} f_{AB}\left(N_{i}^{E}{\mathrm{,N}}_{i}^{S};\Theta_{AB},\Psi \right)+f_{Ab}\left(N_{i}^{E}{\mathrm{,N}}_{i}^{S};\Theta_{Ab},\Psi \right)\\ +f_{aB}\left(N_{i}^{E}{\mathrm{,N}}_{i}^{S};\Theta_{aB},\Psi \right)+f_{ab}\left(N_{i}^{E}{\mathrm{,N}}_{i}^{S};\Theta_{ab},\Psi \right) \end{array}\right] \end{array}$$

where $N_{i}^{E}=\left(N_{i}^{E}\left(1\right),\ldots ,N_{i}^{E}\left(T\right)\right)$ and $N_{i}^{S}=\left(N_{i}^{S}\left(1\right),\ldots ,N_{i}^{S}\left(T\right)\right)$ are the vectors of abundance trajectories at T times for species E and S, respectively, and $f_{\cdot }\left(N_{i}^{E}{\mathrm{,N}}_{i}^{S};\Theta ,\Psi \right)$ is a multivariate normal distribution with expected mean vector (6 parameters) and covariance matrix Σ (5 parameters) for pair i that belongs to a particular genotype–genotype combination. The covariance matrix can be simulated using the second-order structured antedependence (SAD(2)) model (Zimmerman et al., 2001; Zhao et al., 2005).

Based on the dynamic likelihood model (1), it becomes a crucial issue how to map the significant QTLs related to the interaction between two species. We can test whether there are significant interspecific QTLs involved in interspecific interactions, which can be done by formulating the following two assumptions:


(2a) $$H_{0}:\Theta_{AB}=\Theta_{Ab}=\Theta_{aB}=\Theta_{ab}\equiv \Theta$$

(2b) $$H_{1}:\mathrm{Not}\;\mathrm{all}\;\mathrm{equalities in the}\;H_{0}\;\mathrm{hold}$$

where $H_{0}$ is the null hypothesis that each component uses the same ODE parameters in different genotypes. $H_{1}$ is an alternative hypothesis, which means that there is at least one component in the system that has different ODE parameters under different genotypes. Using the values of $H_{0}$ and $H_{1}$ to calculate the likelihood values $L_{0}$ and $L_{1}$ , respectively, and further calculate their log-likelihood ratio (LR), which can be expressed as


(3) $$LR=-2\log\frac{L_{0}}{L_{1}}$$

These LR values are compared to genome-wide critical thresholds determined by 1,000 permutation tests. If $H_{0}$ is rejected, it means that significant interspecific QTLs from both species have been mapped.

### 2.5. Functional annotation

After mapping all the significant QTLs among species, we further implemented the corresponding functional annotations, so as to obtain the key genes that play a critical role in the interaction process of different species in the system, which can provide a theoretical basis for the study of competition and cooperation mechanism among species. Meanwhile, we proofread the results of GO annotation via MGI^1^ and AureoWiki^2^ databases.

### 2.6. Interspecies interaction model

We used a set of generalized Lotka-Volterra (LV) ordinary differential equations to describe the interaction between *E. coli* and *S. aureus* in co-culture (Fujikawa et al., 2014; Jiang et al., 2018a,b). The LV equations were integrated into the systems mapping model, which can screen out significant QTLs for the interaction between the two species in co-culture. We combined this new approach with community ecology so that patterns of interactions between two species can be quantified and explained. The LV equations could be divided into two different parts to describe microbial abundance differently, expressed as


(4) $$\{\begin{array}{c} \begin{array}{l} {\dot{N}}_{E}=r_{E}N_{E}\left(1-\frac{N_{E}+\alpha_{\mathrm{E|S}}N_{S}}{K_{E}}\right)=\\ r_{E}N_{E}\left(1-\frac{N_{E}}{K_{E}}\right)+r_{E}N_{E}\left(\frac{-\alpha_{\mathrm{E|S}}N_{S}}{K_{E}}\right)\equiv {\dot{M}}_{E}+{\dot{N}}_{\mathrm{E|S}} \end{array}\\ \begin{array}{l} {\dot{N}}_{S}=r_{S}N_{S}\left(1-\frac{N_{S}+\alpha_{\mathrm{S|E}}N_{E}}{K_{S}}\right)=\\ r_{S}N_{S}\left(1-\frac{N_{S}}{K_{S}}\right)+r_{S}N_{S}\left(\frac{-\alpha_{\mathrm{S|E}}N_{E}}{K_{S}}\right)\equiv {\dot{M}}_{S}+{\dot{N}}_{\mathrm{S|E}} \end{array} \end{array}$$

where $r_{E}$ and $r_{S}$ are the Malthusian growth rates; $K_{E}$ and $K_{S}$ are the environmental capacities of the two different species; and $\alpha_{\mathrm{E|S}}$ and $\alpha_{\mathrm{S|E}}$ are dimensionless parameters used to simulate how one species affects the other through the interaction in co-culture; ${\dot{M}}_{E}$ and ${\dot{M}}_{S}$ represent the independent growth of each species, which is determined by its inherent attributes; ${\dot{N}}_{\mathrm{E|S}}$ and ${\dot{N}}_{\mathrm{S|E}}$ represent the interactive growth of each species, which is determined by how the species interacts with the other in co-culture. If the interactive growth of a species is positive or negative, it indicates that the species is beneficially or detrimentally affected by the other. If the interactive growth is zero, it means that the two species will not affect each other. Therefore, by estimating ODEs parameters $\Theta =\left(r_{E},K_{E},\alpha_{\mathrm{E|S}};r_{S},K_{S},\alpha_{\mathrm{S|E}}\right)$ , the LV equations can not only specify the dynamic pattern of each species abundance, but also describe two interaction patterns of two species in co-culture.

### 2.7. Quantitative genetic model

According to quantitative genetic theory (Li and Wu, 2009), four interspecific genotypic values can be partitioned into different components and then converted into various genetic effect components, expressed as


(5) $$\begin{array}{l} \left(\begin{array}{c} \mu_{AB}^{l}\left(t\right)\\ \begin{array}{c} \mu_{Ab}^{l}\left(t\right)\\ \mu_{aB}^{l}\left(t\right) \end{array}\\ \mu_{ab}^{l}\left(t\right) \end{array}\right)=\left(\begin{array}{cc} \begin{array}{ccc} 1 & 1 & 1\\ 1 & 1 & -1\\ 1 & -1 & 1 \end{array} & \begin{array}{c} 1\\ -1\\ -1 \end{array}\\ 1\;\;-1\;\;-1 & 1 \end{array}\right)\left(\begin{array}{c} \mu^{l}\left(t\right)\\ \begin{array}{c} a_{A}^{l}\left(t\right)\\ a_{B}^{l}\left(t\right) \end{array}\\ I_{A\times B}^{l}\left(t\right) \end{array}\right)\Rightarrow \\ \left(\begin{array}{c} a_{A}^{l}\left(t\right)\\ a_{B}^{l}\left(t\right)\\ I_{A\times B}^{l}\left(t\right) \end{array}\right)=\frac{1}{4}\left(\begin{array}{ccc} 1 & 1 & \begin{array}{cc} -1 & -1 \end{array}\\ 1 & -1 & \begin{array}{cc} 1 & -1 \end{array}\\ 1 & -1 & \begin{array}{cc} -1 & 1 \end{array} \end{array}\right)\left(\begin{array}{c} \mu_{AB}^{l}\left(t\right)\\ \begin{array}{c} \mu_{Ab}^{l}\left(t\right)\\ \mu_{aB}^{l}\left(t\right) \end{array}\\ \mu_{ab}^{l}\left(t\right) \end{array}\right) \end{array}$$

Where $\mu_{AB}^{l}\left(t\right),\mu_{Ab}^{l}\left(t\right),\mu_{aB}^{l}\left(t\right)\;\mathrm{and}\;\mu_{ab}^{l}\left(t\right)$ are the genotypic values for the abundance of each species l(l=EorS) at time t ; $\mu^{l}\left(t\right)$ is the time-dependent population means of abundance of each species l(l=EorS) ; if l=E , $a_{A}^{l}\left(t\right)$ is the time-dependent direct genetic effect of species E’s QTL on its own abundance, while if l=S , $a_{A}^{l}\left(t\right)$ is the time-dependent indirect genetic effect of species E’s QTL on the abundance of its coexisting species S; if l=S , $a_{B}^{l}\left(t\right)$ is the time-dependent direct genetic effect of species S’s QTL on its own abundance, while if l=E , $a_{B}^{l}\left(t\right)$ is the time-dependent indirect genetic effect of species S’s QTL on the abundance of its coexisting species E; and $I_{A\times B}^{l}\left(t\right)$ is the time-dependent genome–genome epistatic effect between the QTLs of two species on the abundance of each species l(l=EorS) .

After mapping significant interspecies QTLs, the next step is to test whether these QTLs exert significant direct genetic effects, indirect genetic effects, and interspecies genome-genome epistasis effects. The null hypotheses for these tests are expressed as


(6a) $$H_{0}:a_{A}^{E}\left(t\right)=0\;\mathrm{and}\;a_{B}^{S}\left(t\right)=0\;\mathrm{for direct effects}$$

(6b) $$H_{0}:a_{A}^{S}\left(t\right)=0\;\mathrm{and}\;a_{B}^{E}\left(t\right)=0\;\mathrm{for indirect effects}$$

(6c) $$\begin{array}{l} H_{0}:I_{A\times B}^{E}\left(t\right)=0\;\mathrm{and}\;I_{A\times B}^{S}\left(t\right)=\\ \;\;\;\;\;\;\;\;\;\;0\;\mathrm{for genome}-\mathrm{genome epistatic effects} \end{array}$$

### 2.8. Quantitative trait loci networks

For a given QTL, the systems mapping models can estimate its time-dependent direct genetic effects on independent growth, time-dependent indirect genetic effects on interactive growth, and time-dependent genome-genome epistasis effects on interactive growth. The procedure of constructing the corresponding genetic network of all QTLs based on their genetic effects is as follows: Let $q_{m}\left(t\right)$ denotes the genetic effects of QTL m(m=1,…,M) at time t . All these M QTLs interact with each other in a network to jointly affect growth. Through a set of ordinary differential equations, $q_{m}\left(t\right)$ is used to denote the genetic effects of other QTLs, expressed as


(7) $${\dot{q}}_{m}\left(t\right)=w_{m}\left(q_{m}\left(t\right)\right)+\overset{M}{\underset{m^{\prime }=1,m^{\prime }\neq m}{\sum }}u_{m^{\prime }}\left(q_{m^{\prime }}\left(t\right)\right)$$

where $w_{m}\left(q_{m}\left(t\right)\right)$ is the parametric or non-parametric function of $q_{m}\left(t\right)$ , and $u_{m^{\prime }}\left(q_{m^{\prime }}\left(t\right)\right)$ is the parametric or nonparametric function of the genetic effects of QTL $m^{\prime }\left(m^{\prime }\neq m,m^{\prime }=1,\ldots ,M\right)$ . The latter presents the interactive effects of other QTLs on the focal QTL. Non-parametric methods (Wu et al., 2014) are used to estimate the parameters of these functions, so that the corresponding genetic effect network can be constructed among these QTLs, and how a QTL interacts with others through activation or inhibition, thereby affecting the expression of the latter.

## 3. Results

### 3.1. Analysis of population structure and kinship relationship

According to the marginal possibility, we identified eight and nine subpopulations in *E. coli* and *S. aureus* strains, respectively (Supplementary Figure S1A). In terms of PCA analysis, the first two PCs of *E. coli* accounted for 50.4 and 11.5% of the variation in the genotypic data respectively, and PC1 and PC3 of *S. aureus* accounted for 47.5 and 10.0%, respectively (Supplementary Figure S1B). The results of fastStructure and PCA indicated significant population structure differences among individuals of the natural population of these two bacteria. The confounding effects of population structure and kinship were well removed to a certain extent (Supplementary Figures S1C, S2, S3). The adjusted phenotypic data can be used for systems mapping of the two species in co-culture.

### 3.2. Mapping significant QTLs for the interaction of two bacteria in co-culture

**Figure 1:**
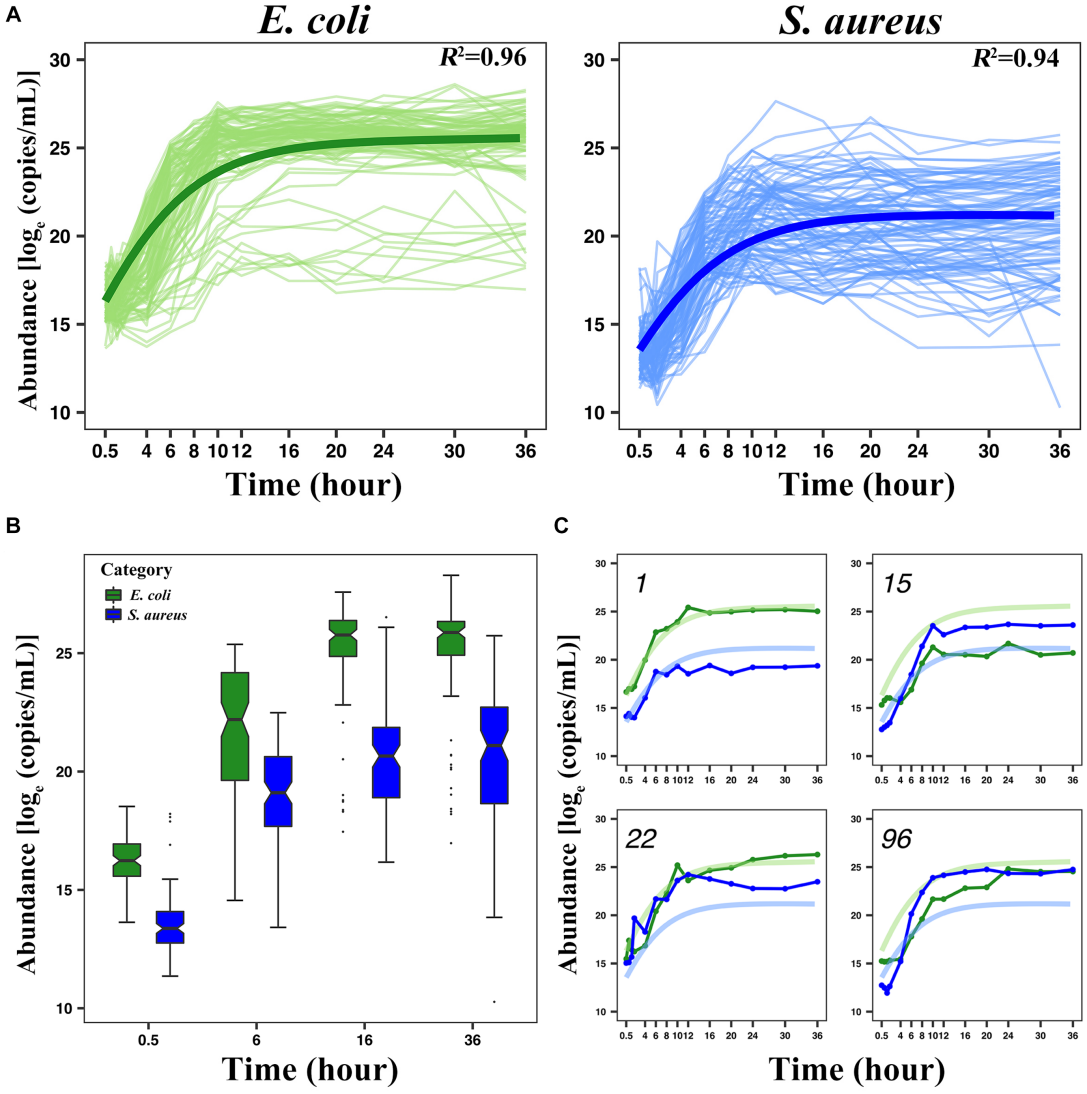
Analysis of the growth trajectory of *E. coli* and *S. aureus*. **(A)** The average growth curve of 100 pairs of *E. coli* and *S. aureus*, in which both of the coefficients of variation were greater than or equal to 0.94. **(B)** The boxplot displaying the phenotypic distribution of *E. coli* and *S. aureus* at selected 4-time points (0.5 h, 6 h, 16 h, and 36 h). **(C)** Four pairs of representative individual growth curves from 100 pairs of combinations (1st, 15th, 22nd, and 96th pairs). The green color indicates *E. coli* and the blue color indicates *S. aureus*. In this part label **C**, the light-colored curves represent the average growth curve, and the dark-colored curves represent the actual growth curve.

Taking the number of microbes (the number or weight of the living bacteria) as the vertical coordinate, and the cultivation time as the horizontal coordinate, we can draw a microbial growth curve. A typical microbial growth process includes three successive growth phases: lag, logarithmic, and stationary phases. At the lag phase, the population adapts to a new environment, in which the specific growth rate gradually increases from zero and then accelerates the maximum value at the logarithmic phase. At the stationary phase, however, the rate decreases to zero and the population growth terminates, thereby reaching the culture-carrying capacity. A number of growth equations have been derived to capture these phases (Zwietering et al., 1990; Tonner et al., 2020). In this study, a set of generalized LV ordinary differential equations was used to fit the growth curve of *E. coli* and *S. aureus* in co-culture during the first 36 h after culture (Figure 1A). For the growth curve of each strain, the actual individual growth curve and the fitted growth curve of 100 pairs of interspecific strains of *E. coli* and *S. aureus* were shown in Supplementary Figures S4, S5. In the average growth curve, we can observe that the fitting of the two bacteria met the analysis requirement, with a coefficient of variation *R^2^* ≥ 0.94, which indicated that the growth curve fitted by LV can well represent the dynamic phenotype data of the interaction between the two bacteria. Overall, the microbial abundance of *E. coli* in the unit time is generally higher than *S. aureus*. For example, at 0.5 h, the abundance value of *E. coli* is over $e^{16}$ , while that of *S. aureus* is less than $e^{14}$ . Selecting 0.5 h, 6 h, 16 h, and 36 h to draw the phenotype distribution of the two bacteria (Figure 1B), we could see that the vast majority of the *E. coli* strains grow faster than *S. aureus* strains, but there were also some outliers indicating the existence of some opposite situations. We selected four kinds of representative growth curves (Figure 1C) and found that, in addition to the fact that *E. coli* has consistently been leading the growth of *S. aureus* or vice versa, there were also two bacteria with similar initial growth conditions, but during the logarithmic period, the growth of *E. coli* exceeded that of *S. aureus* and even *S. aureus* was severely inhibited. This indicates that there may be some genes that affect the actual growth of the two bacteria when they interact, and the degree of influence varies greatly between different bacterial pairs.

**Figure 2:**
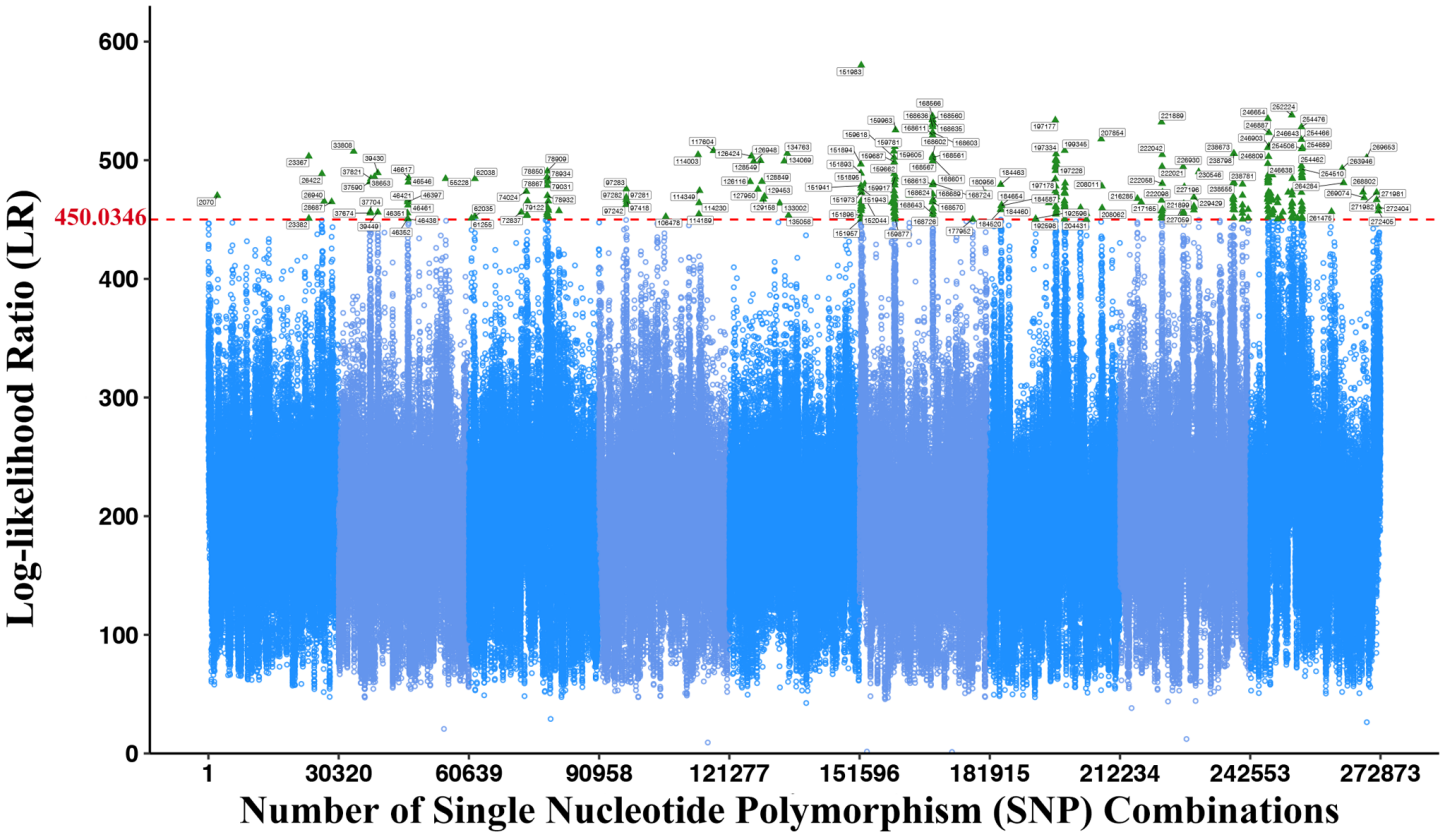
Manhattan plot displaying the systems mapping result of significant interspecific QTLs in 272,873 SNP combinations. The green triangle indicates the significantly associated SNP combinations. The red dashed line indicates the threshold with 1,000 permutation tests at 450.0346.

The correlation analysis between the phenotypic data of two bacteria in co-culture and SNP genotype data by using the systems mapping theory can excavate the QTL closely related to the interspecific interaction. The LR threshold at the genome-wide level was determined by 1,000 permutation tests, and we could draw the Manhattan diagram (Figure 2) and search out significant QTL combinations affecting the microbial abundance through the interspecific interaction, whose LR values are greater than the LR threshold. The LR threshold of this study was 450.0346, and 244 vital QTL combinations were obtained through comparison, including 69 SNPs from *E. coli* and 59 SNPs from *S. aureus* (Supplementary Tables S2, S3). These SNPs were further mapped to genes, and 57 and 43 genes were obtained in *E. coli* and *S. aureus*, respectively (Supplementary Tables S4, S5).

### 3.3. Functional annotation and interpretation of significant interspecific interaction QTLs

Based on the results of 3.1, we successfully annotated 57 genes in *E. coli* with 44 effective GO annotation results and 43 genes in *S. aureus* with 38 effective GO annotation results. Supplementary Tables S4, S5, respectively, listed the detailed functional annotation information of the significant interspecific interaction QTLs of the two bacteria.

**Figure 3:**
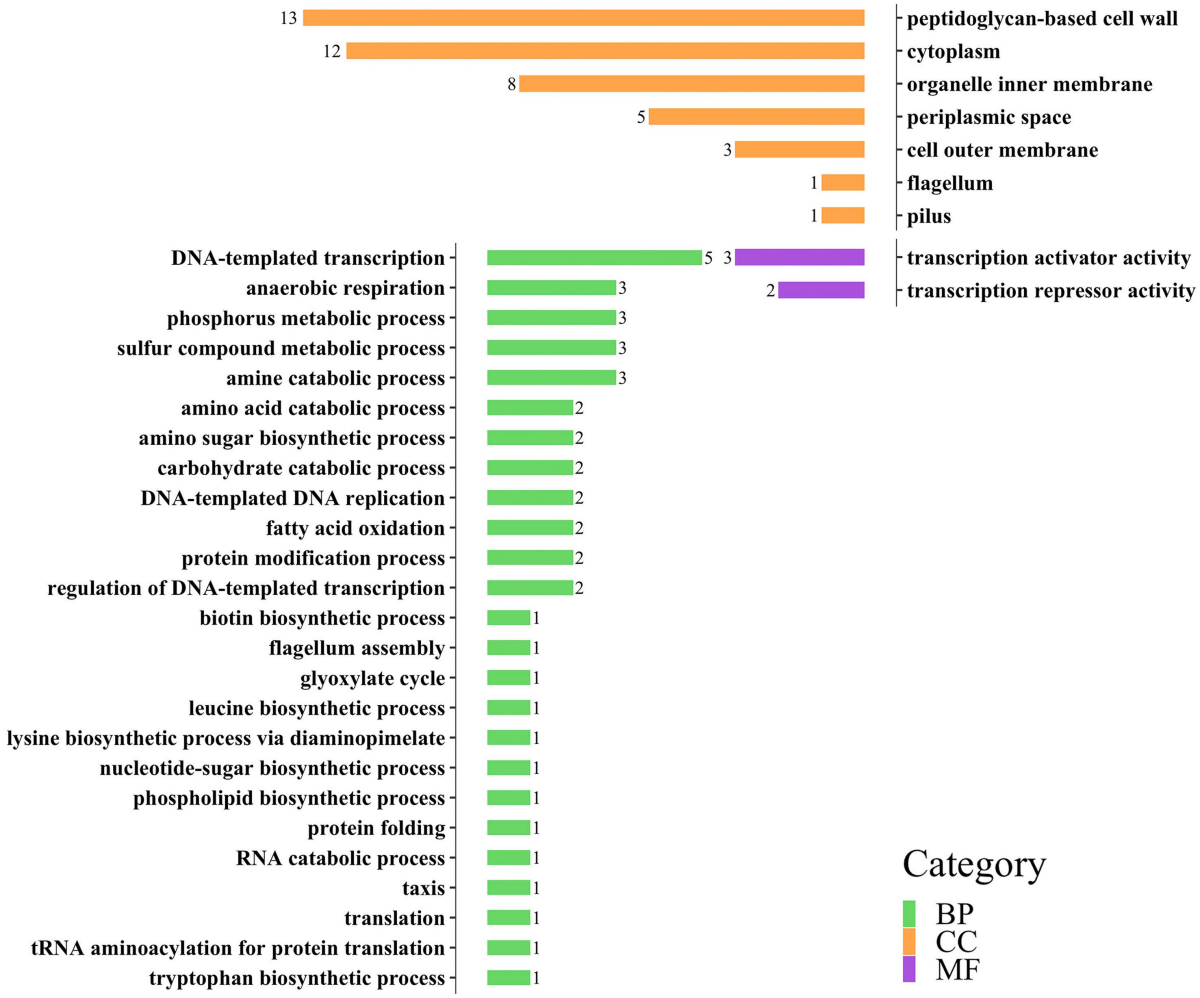
Distribution of three categories of GO annotation results of *E. coli*. The green color represents BP, the orange color represents CC, and the purple color represents MF.

**Figure 4:**
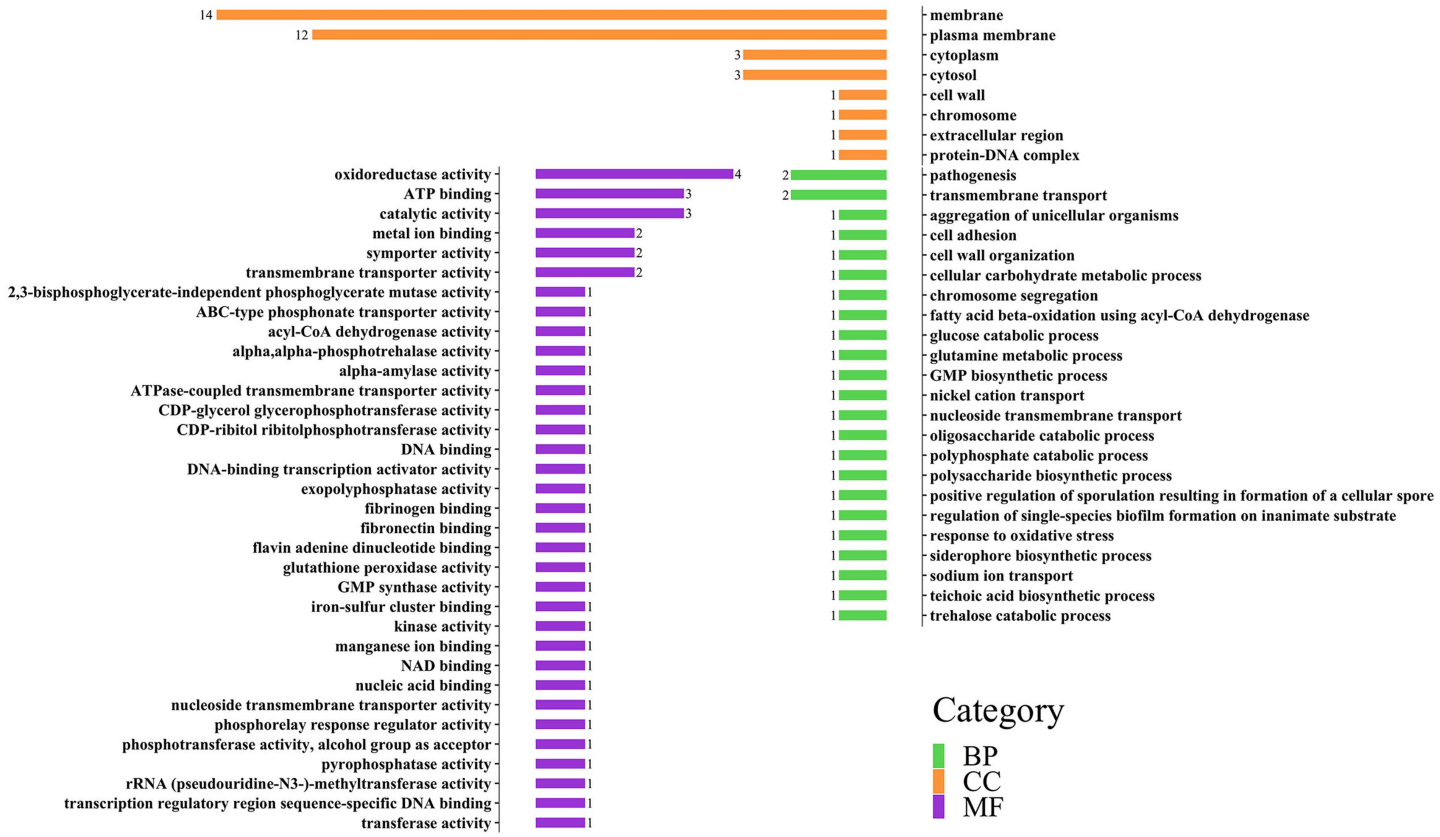
Distribution of three categories of GO annotation results of *S. aureus*. The green color represents BP, the orange color represents CC, and the purple color represents MF.

GO annotations can be divided into three categories: Biology Process (BP), Cellular Component (CC), and molecular function (MF). Each large category can be subdivided into subcategories, which can help us understand the biological significance behind each gene (Figures 3, 4).

In the gene annotation of *E. coli* (Figure 3), the BP category involved 37 genes, accounting for the highest proportion (65%), followed by the CC category (31 genes, 54%). The BP category mainly included DNA-templated transcription (5 genes), anaerobic respiration (3 genes), phosphorus metabolic process (3 genes), and sulfur metabolic process (3 genes); the CC category mainly included peptidoglycan-based cell wall (13 genes), cytoplasm (12 genes), and organelle inner membrane (8 genes); the MF category includes transcription activator activity (3 genes) and transcription repressor activity (2 genes).

In the gene annotation of *S. aureus* (Figure 4), the CC category involved 29 genes, accounting for the highest proportion (67%), followed by the MF category (23 genes, 53%). The CC category was mainly related to membranes, such as membrane (14 genes) and plasma membrane (12 genes); the MF category included oxidoreductase activity (4 genes), ATP binding (3 genes), and catalytic activity (3 genes); the BP category mainly included pathogenesis (2 genes) and transmembrane transport (2 genes).

### 3.4. Modeling the genotype–phenotype relationship

Any species in co-culture may choose to cooperate or compete with other species from the same system, depending on the level at which common resources can be shared for their respective growth. According to the community ecology theory, the interaction types of two different species can be formulated by a strategy matrix (Supplementary Figure S6), including mutualism, competition, neutralism, commensalism, predation/parasitism, and amensalism.

Different strategies used between two species in one system resulted in six distinct types of interactions: (++) → Mutualism, which means that two species benefit from each other; (00) → Neutralism, which means that any one species does not depend on or affect the other; (−-) → Competition, which means that two species fight each other; (+0) → Commensalism, which means that one species is beneficial to the other, while the latter does not affect the former; (+−) → Predation/Parasitism, which means that one species helps the other, whereas the latter is harmful to the former; (−0) → Amensalism, which means that one species harms the other, while the latter does not affect the former.

**Figure 5:**
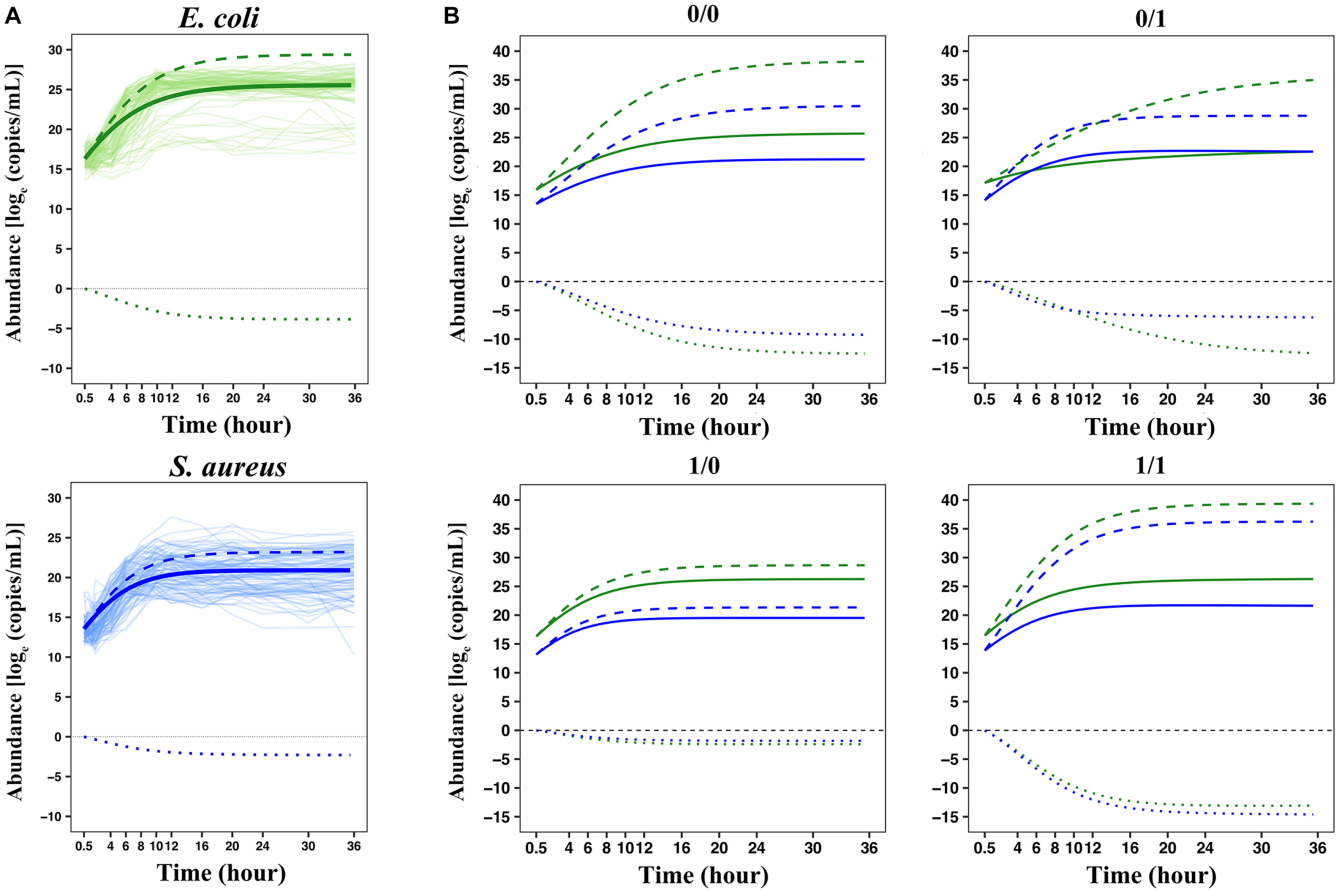
The interspecies interaction pattern of two bacteria in co-culture based on the LV equations. **(A)** Overall growth, independent growth, and interactive growth curves of two bacteria in co-culture. **(B)** Genotypic differences in the growth curves of the two bacteria. Four genotype–genotype combinations 0/0, 0/1, 1/0, and 1/1 at the QTL combination E3414424 × S383182 were fitted by a set of generalized LV ordinary differential equations. The green lines represent *E. coli* and the blue lines represent *S. aureus*. The solid lines represent the overall growth curves fitted to each set of actual observed data, decomposed into independent growth curves (long dashed lines) and interactive growth curves (short dashed lines).

Based on the LV equations, the overall growth of each species in co-culture can be decomposed into independent growth and interactive growth. By estimating the ODEs parameters $\Theta =\left(r_{e},K_{e},\alpha_{\mathrm{e|s}};r_{s},K_{s},\alpha_{\mathrm{s|e}}\right)$ , we can characterize the interaction mode of two species in co-culture by comparing the interactive growth between species. We plotted the overall growth curves (solid lines), independent growth (long dashed lines), and interactive growth curves (short dashed lines) of *E. coli* and *S. aureus* over time in co-culture, according to ODE parameters (Figure 5A). On the whole, the interactive growth was always negative because two bacteria in the same environment compete with each other for limited resources, and the independent growth is much greater than the actual overall growth observed, indicating that these two bacteria have a competitive relationship in co-culture.

Each interspecies QTL combination inherited two bacterial alleles 0 and 1, respectively, forming four interspecies genotype–genotype combinations, denoted as 0/0, 0/1, 1/0, and 1/1. Taking the selected QTL combination E3414424 × S383182 (LR value = 505.2964) as an example to analyze the interaction between two bacteria in co-culture, we drew the microbial growth curves of four genotype combinations of two bacteria in co-culture (Figure 5B). By combining the relevant parameters of ODEs, the overall growth curve (solid line) of microbial abundance for each genotype combination can be decomposed into independent growth (long dashed line) and interactive growth curve (short dashed line). For each genotype combination, it can be observed that the competition between *E. coli* and *S. aureus* was intense, and the overall growth was lower than the independent growth, but the overall growth, independent growth, and interactive growth were significantly different in scale and pattern among all four combinations. These results can be summarized as follows: (1) The two bacteria in the same system were in an antagonistic relationship, but the degree of antagonism of *S. aureus* to *E. coli* was greater than that of *E. coli* to *S. aureus*; (2) When the same genotype was paired, the antagonistic was expressed more strongly, for example, 0/0 and 1/1 exhibited larger negative interactive growth than 0/1 and 1/0; (3) Combination-dependent differences in independent growth and interactive growth was more pronounced than in overall growth, suggesting that bacteria exhibited more variation in their internal machinery than what can be phenotypically observed. These results suggested that E3414424 and S383182 were antagonistic QTLs that participated in determining and shaping the antagonistic relationship between *E. coli* and *S. aureus* when they were in the same system.

### 3.5. Decomposing genetic effects

Interspecies QTL combinations can affect the independent growth of two bacteria as a growth potential and also affect their interactive growth determined by interacting with other species in the ecosystem. To some extent, such QTLs play a radical role in regulating microbial communities’ dynamic structure and behavior. The systems mapping model can also dissect the genetic structure of community traits into its direct, indirect, and genome-genome epistatic genetic effects. The direct genetic effect of an interspecific QTL combination describes how it affects the independent growth of one species (such as *E. coli*) where this QTL is mapped. The indirect genetic effect specifies the genetic effect of an interspecific QTL combination from one species (such as *S. aureus*) on the interactive growth of the other (such as *E. coli*) competing with this species in the same system. And the genome-genome epistatic effect depicts the genetic effect of the interaction between the alleles of two species on the interactive growth of one species.

**Figure 6:**
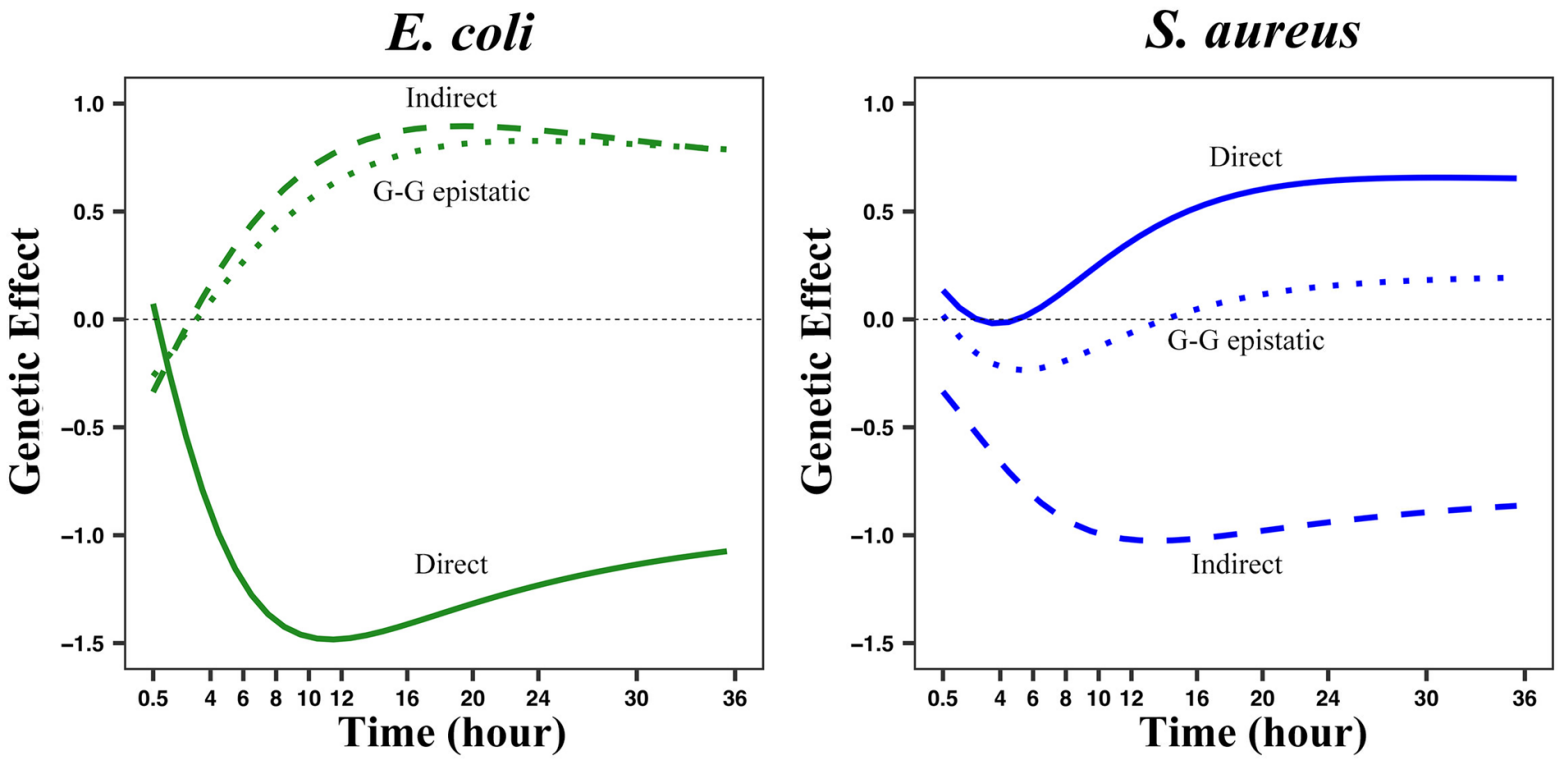
The three genetic effect modes of the growth curve of two bacteria in co-culture were analyzed by QTL combination E3414424 × S383182. The genotypic value of each genotype combination was partitioned into its direct (solid line), indirect (long dotted line), and genome-genome (G-G) epistatic effects (short dotted line) on the growth of each species.

After estimating and mapping these three genetic effects of the interspecies QTL combination (E3414424 × S383182) on the growth trajectory of two bacteria, it was found that the indirect effects and genome-genome epistasis effects, which were ignored in previous studies, were significant, and even their influence range was greater than the direct effect (Figure 6). E3414424 from *E. coli* appeared to be a more “aggressive” QTL, because its indirect effect on the abundance dynamics of *S. aureus* was greater than that of S383182 from *S. aureus* on the abundance dynamics of *E. coli*. Meanwhile, its direct effect on the abundance of its home species at the logarithmic phase was greater than the indirect effect of S383182 on its abundance. From the analysis of the genetic effect curve, we can see how an SNP affects the growth of two microbes over time. For this particular QTL combination, the direct, indirect, and genome-genome epistatic effects on *E. coli* reached their maximum at 10–14 h, and these effects on *S. aureus* showed a similar pattern.

### 3.6. Construction of the genetic effect networks of significant interspecific QTLs

**Figure 7:**
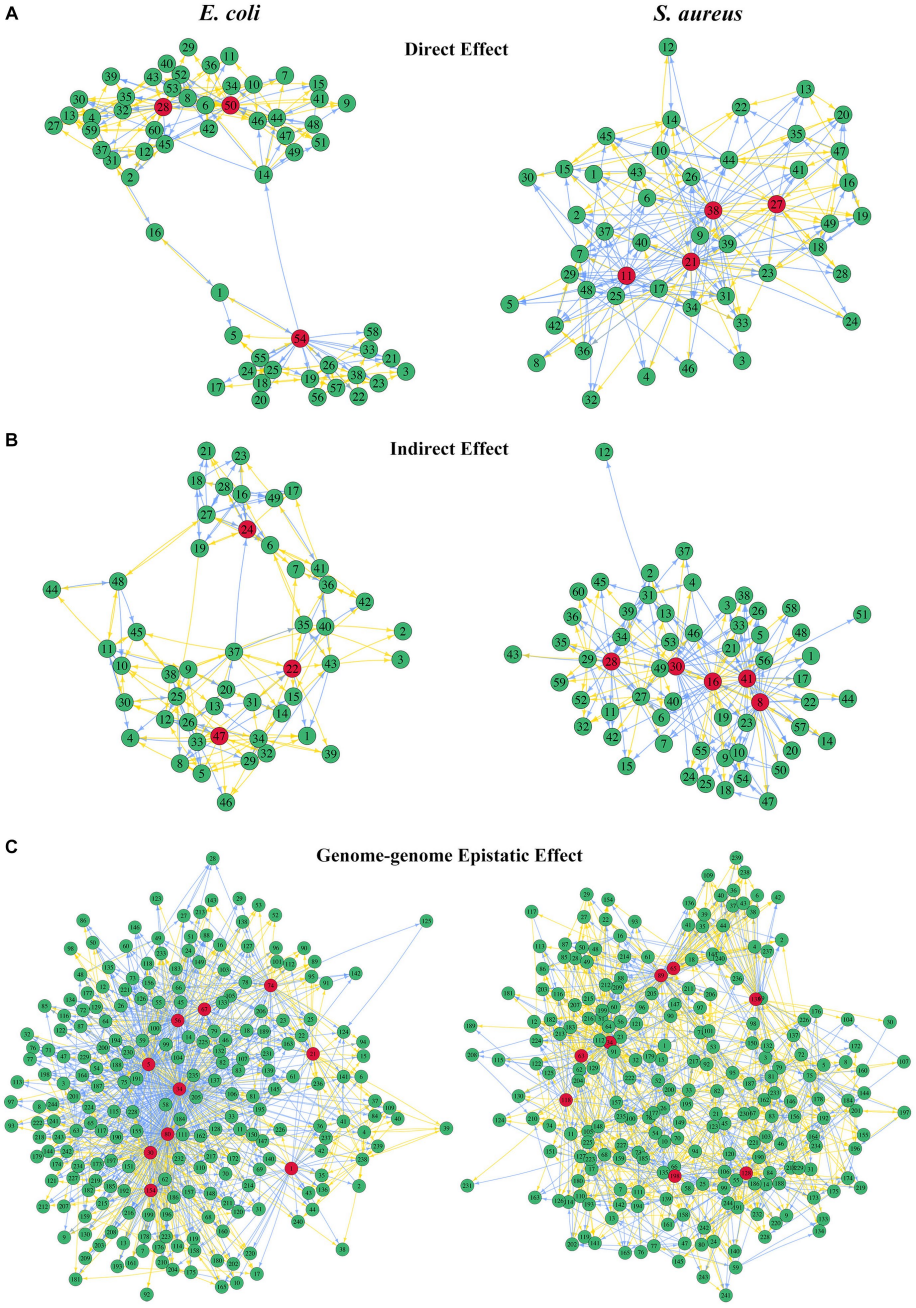
QTL genetic networks through three types of genetic effects. The left figures show the direct effects of 60 *E. coli* QTLs **(A)**, indirect effects of 49 *S. aureus* QTLs **(B)**, and genome-genome epistatic effects of 230 QTL combinations **(C)** on the growth trajectory of *E. coli*. The right figures show the direct effects of 49 *S. aureus* QTLs **(A)**, indirect effects of 60 *E. coli* QTLs **(B)**, and genome-genome epistatic effects of 230 QTL combinations **(C)** on the growth trajectory of *S. aureus*. The hub QTLs in each network are highlighted in red. Arrows indicate the direction of one QTL/effect to another QTL/effect, wherein the orange arrow indicates promotion and the blue arrow indicates inhibition. Names of genes corresponding to each number are given in Supplementary Table S6.

The bacterial abundance of *E. coli* in co-culture was jointly determined by the direct effects of its own 60 QTLs, the indirect effects of 49 QTLs from *S. aureus*, and the interspecific epistatic effects between 230 QTL combinations. The bacterial abundance of *S. aureus* in the same system was determined by the direct and indirect effects triggered by its own 49 QTLs and 60 *E. coli* QTLs, respectively, and the interspecific epistatic effects among 230 QTL combinations. We employed an ODE-based approach to map genetic networks to characterize how these significant QTLs interacted in the network to affect the microbial abundance through three different types of effects (Figure 7). By comparing the three types of QTL networks, we found that the structure and organization of these networks differed greatly between the two bacteria. The core feature of the QTL network is its ability to identify the hub QTLs, which play a vital role in the genetic architecture of microbial growth in the same system.

For *E. coli*, there were a major hub QTL SNP 50 (E4323969) and two minor hub QTLs SNP 28 (E2357245) and 54 (E4544464) in the direct effect network that directly affected its own growth (Figure 7A), while *S. aureus*’s direct effect network had SNP 21 (S301259), 38 (S590022), 11 (S134253), and 27 (S321552) as the hub QTLs directly affecting its own growth. In the indirect effect network, there were 3 hub QTLs from *S. aureus* which exerted an indirect effect on the growth performance of *E. coli*, including SNP 47 (S1731046), 22 (S301667), and 24 (S306067). However, there were 5 hub QTLs from *E. coli* in the network that indirectly affected the growth performance of *S. aureus*, including SNP 16 (E2015702), 41 (E4125943), 8 (E555930), 30 (E2683642), and 28 (E2357245; Figure 7B). Interestingly, whether directly affecting its own growth or being indirectly affected by competing strains, the number of hub QTLs in *S. aureus* was greater than that in *E. coli*. SNP 28 (E2357245) from *E. coli* acted as a hub QTL in both direct-to-self and indirect-to-*S. aureus* effect networks, although the network structures of these two effects were very different. In terms of the genome-genome epistasis effect network, *E. coli* had 10 QTL combinations acting as hubs, and *S. aureus* had 8. Surprisingly, the 34th QTL combination (E2015702 × S1385806) acted as a major hub in *E. coli*, whereas it was a minor hub for *S. aureus* (Figure 7C).

## 4. Discussion

Interactions between species are important drivers of structuring species communities. In biological communities, a member interacts with other members of the same species through density-dependent regulation, and also with members of other species, including mutualism, competition, neutralism, commensalism, predation/parasitism, or amensalism (Ovaskainen et al., 2017). Despite increasing interest in how evolution affects ecological dynamics, most studies of evolutionary adaptation consider single species in isolation (Lawrence et al., 2012). The microbiota is widely recognized as a vital determinant of various natural processes, from biogeographical cycles to human health. Discovering and characterizing mechanisms of microbial interactions can help us reveal a wealth of new biological information about microbes and can provide insights into how the microbiome can be manipulated to improve medical, agricultural, and environmental relationships (Pierce and Dutton, 2022). For instance, predict the stability of intestinal flora (Fang et al., 2021), resist the infection of pathogenic bacteria (Levy and Borenstein, 2013), degrade complex organic matter in the deep-sea environment (Nawaz et al., 2022), and optimize the design of microbial engineering (Perez-Garcia et al., 2016).

The development of next-generation sequencing technology has made it possible to explore the interaction of complex microbial communities or microbiomes, although there are many challenges and limitations (Carr et al., 2019). It is very difficult to observe microbial interactions *in situ*, while theoretical model research can set the mechanism or parameters of microbial interactions, which is a flexible and powerful tool for exploring the ecology of microbial community ecology. Current research on the mechanism of microbial interactions, mainly including interactions related to toxic molecules, nutrient competition and cross-feeding, access to metals, signaling pathways, pH changes, and interactions within biofilms (Pierce and Dutton, 2022), has rarely deeply explored which QTLs play a key role in the microbial interaction process at the molecular level. Although it has been documented that conventional mapping approaches can map QTLs responsible for interspecies interactions in terms of the immune response, tolerance to herbivores, mate recognition, or predator-defense traits, they cannot distinguish phenotypic independent and interacting components, but focus directly on the observed overall phenotype (Jiang et al., 2018a). Embedding community ecology into the genetic mapping model has been proven to be very effective and powerful for studying the genetic landscaping of how plants coexist (Jiang et al., 2018a) and the mechanisms of microbial interactions (Jiang et al., 2018b). In this study, we designed an ecological experiment for 100 pairs of *E. coli* and *S. aureus* in co-culture and applied the systems mapping model based on the generalized LV interspecific competition equations to study the interaction between these two bacteria. Our model can decompose the overall phenotype into independent and interactive parts, and quantify the genetic effects between these two bacteria into direct effects, indirect effects, and genome-genome epistatic effects, so as to analyze the mechanism of competition and cooperation between the two species in the same environment and provide a new perspective to solve the problem of missing heritability.

Systems mapping is a dynamic model, which capitalizes on time series phenotypic data to search for interspecific interaction QTLs. According to the results of systems mapping, we have counted and drawn the frequency distribution of the interspecific interaction genes mapped between *E. coli* and *S. aureus* (Supplementary Figure S7). Among them, the significant interspecific QTLs from *E. coli* were mainly mapped on 7 genes, including *ypdC* (23, 10.00%), *nrfC* (23, 10.00%), *yphH* (21, 9.13%), *acrE* (19, 8.26%), *dcuS* (17, 7.39%), *rpnE* (12, 5.22%), and *ptsA* (11, 4.78%). These genes were functionally annotated for more information. YpdC is annotated as an AraC-type regulator with a C-terminal helix-turn-helix (HTH) domain, so it may have regulatory functions under the appropriate growth conditions (Gao et al., 2021). NrfD-like proteins can associate with NrfC-like FeS proteins to form a dimeric redox module involved in quinone redox chemistry, so as to provide the proton or sodium motive force required for ATP synthesis in prokaryotes (Duarte et al., 2021). The fumarate, or C4-dicarboxylate (C4DC), responsive sensor kinase DcuS of *E. coli* is anchored by TM helices TM1 and TM2 in the membrane, which as extra-cytoplasmic sensor domain is related to transmembrane (TM) signaling (Stopp et al., 2021). The *ptsA* gene is related to inorganic ion transport and metabolism (Song et al., 2018) and is involved in phosphate and nickel transport, which may represent genes responsible for adaptation to stress or other environmental signals (Spoto et al., 2022). In terms of *S. aureus*, the significant interspecific QTLs were mainly mapped on 7 genes, including *ebh* (38, 16.52%), *SAOUHSC_00172* (37, 16.09%), *capF* (23, 10.00%), *gdpP* (12, 5.22%), *orfX* (11, 4.78%), *bsaA* (11, 4.78%), and *phnE1* (10, 4.35%), which were also functionally annotated for more information. CapF enzyme can catalyze the synthesis of UDP-N-acetyl-L-fucosamine, which is a component of capsular polysaccharide, an important virulence factor of *S. aureus* (Miyafusa et al., 2008). Mutations in *gdpP* are significantly related to meticillin-resistant lacking *mec* (MRLM) phenotype, and its encoded GdpP is a phosphodiesterase, which participates in the degradation of cyclic-di-AMP, the second messenger in *S. aureus* (Sommer et al., 2021). The gene *orfX* is conserved among all staphylococci, whose product has been suspected to play an important role in bacterial growth and survival (Boundy et al., 2013). The mutant *S. aureus* expressing AgrAC199S was more susceptible to H_2_O_2_ due to repression of the antioxidant *bsaA* gene under oxidative stress, and this oxidation sensing could serve as an intrinsic checkpoint to ameliorate the oxidation burden caused by intense metabolic activity and potential host immune response (Sun et al., 2012). A study using *phnE1* deletion strains confirmed that low phosphate (P_i_) concentrations in the media could increase the uptake of phosphorylated amino acids and could divert or inhibit their subsequent breakdown into P_i_ to further stabilize the levels of the phosphorylated amino acid, which can ensure P_i_ is provided to the intracellular environment and made available for vital processes (Steinfeld et al., 2014). The gene information provides a certain reference for further exploring the genetic mechanism of the interaction between the two bacteria, especially in experimental verification, and more specific pathway information needs to be further explored.

By decomposing the overall phenotype based on the LV equations, we can estimate how QTLs regulate interspecific competition and cooperation and interpret the vital role of these QTLs in organizing community structure and function through six patterns. We detected the QTL combination to analyze the interaction between two bacteria in co-culture, including the gene *acrE* located at E3414424 position in *E. coli* and the gene *guaA* located at S383182 position in *S. aureus*. By analyzing the growth curve of the four genotypes for this QTL combination (Figure 5C), we found that most genotypes were in a competitive relationship, to compete for limited medium resources and space for survival. The *acrE* gene encodes multidrug efflux pump membrane fusion lipoprotein AcrE, which can recognize a variety of toxic chemicals and actively expel them from cells. It acts on a wide range of substrates, ranging from most antibiotics, disinfectants, dyes, and detergents to simple solvents (Ma et al., 1993; Lu et al., 2006; Alav et al., 2021). The *guaA* gene encodes guanosine monophosphate (GMP) synthase. GMP is a vital cellular metabolite for signal transduction (e.g., cyclic di-GMP) as well as bacterial virulence and survival (Hall and Lee, 2018). GMP can be directly generated from guanine and guanosine through a simple enzymatic reaction, but the inactivation of *guaA* generally results in guanine auxotrophy, therefore, *guaA* is essential for the *de novo* biosynthesis of GMP (Kotloff et al., 2004; Kofoed et al., 2016; Smith-Peter et al., 2021). This illustrates that our model can glean new insights into the genetic architecture of interspecific interactions, species coevolution, and community dynamics.

Our model could visualize three kinds of genetic effect networks, which will be particularly useful for inferring and predicting the genetic mechanisms of community dynamics and evolution through further mining hub QTLs of these networks. Interestingly, *yphH* (E2683642), *rpnE* (E2357245), and *ebh* (S1385806) genes were not only genes with high frequency in interspecific QTL mapping, but also hub genes in the genetic effect networks. The *yphH* gene as harboring microdiversity can help bacteria adapt to varying and challenging environments by modifying their surface proteins (Touzain et al., 2010). Bacteria use a variety of DNA-mobilizing enzymes to facilitate environmental niche adaptation via horizontal gene transfer, and a study found that *rpnA-E* genes of *E. coli* encode nucleases involved in DNA recombination, but overexpression of RpnA (YhgA) to RpnD (YjiP) increased RecA-independent recombination, reduced cell viability, and induced the expression of reporter of DNA damage, while RpnE (YfaD) is inactive in these processes (Kingston et al., 2017). The *ebh* gene is one of the mutations with possible influence on resistance phenotype identified in the genome of *S. aureus* SG511 (Dietrich et al., 2021), and mutations that disrupt the *ebh* reading frame are associated with increased oxacillin and teicoplanin susceptibility (Cheng et al., 2014). In particular, the *rpnE* gene is a hub gene in the direct genetic effect network of *E. coli* and the indirect genetic effect network of *S. aureus*, indicating that this gene plays a pivotal role in the process of microbial interspecies interaction, and is worth further exploring its function and significance.

The application of systems mapping advances our understanding of microbial community structure and function, and also provides guidance for the efficient design of related experiments. Theoretical model studies provide new insights into microbial community ecology, while experimental validation of microbial interactions compensates for the limitations of model inference. In the future, the combination of systems mapping and gene function verification will not only deepen the understanding of the mechanism of microbial interspecies interactions, but also improve the ability to predict the dynamics and functional changes of microbial communities, and then further respond to challenges such as invasive alien species and global climate change, by regulating the species composition and interaction patterns of microbial communities.

## Data availability statement

The datasets presented in this study can be found in online repositories. The names of the repository/repositories and accession number(s) can be found below: NCBI–*E. coli* 1-100: https://ngdc.cncb.ac.cn/gsa/browse/CRA010512 *S. aureus* 1-100: https://ngdc.cncb.ac.cn/gsa/browse/CRA010516.

## Author contributions

RW and XZ conceived the idea and designed the model. XH and YJ designed the experiments. CL and LY performed the experiments. CL performed data analysis and wrote the manuscript. XZ provided advice and guidance on the manuscript. All authors contributed to the review and approval of the current version of the manuscript.

## Footnotes

^1^ https://www.informatics.jax.org/vocab/gene_ontology

^2^ https://aureowiki.med.uni-greifswald.de/Main_Page
