# Supplementary material for: Competition-cooperation mechanism between Escherichia coli and Staphylococcus aureus based on systems mapping
Source: Front Microbiol. 2023 Nov 6;14:1192574. doi: 10.3389/fmicb.2023.1192574 (PMC10657823; doi:10.3389/fmicb.2023.1192574)
Supplement: Supplementary file 2 [file Data_Sheet_1.docx]

Supplementary Material

Competition-cooperation mechanism between *Escherichia coli* and *Staphylococcus aureus* based on systems mapping

Caifeng Li^1^, Lixin Yin^1^, Xiaoqing He^1,2,3,4^, Yi Jin^1,2,3,4^, Xuli Zhu^1,2,3,4^*, Rongling Wu^1,2,3,4^

*** Correspondence:** Xuli Zhu: xulizhu@bjfu.edu.cn

# Supplementary Figures and Tables

## Supplementary Figures





**Supplementary Figure 1.** Population structure analysis of *E. coli* and *S. aureus*. **(A)** Distruct plot of faststructure analysis for two bacteria, where each color represents a subpopulation. **(B)** PCA analysis of two bacteria, where different colored dots represent different subpopulations. **(C)** Population structure residual plots.


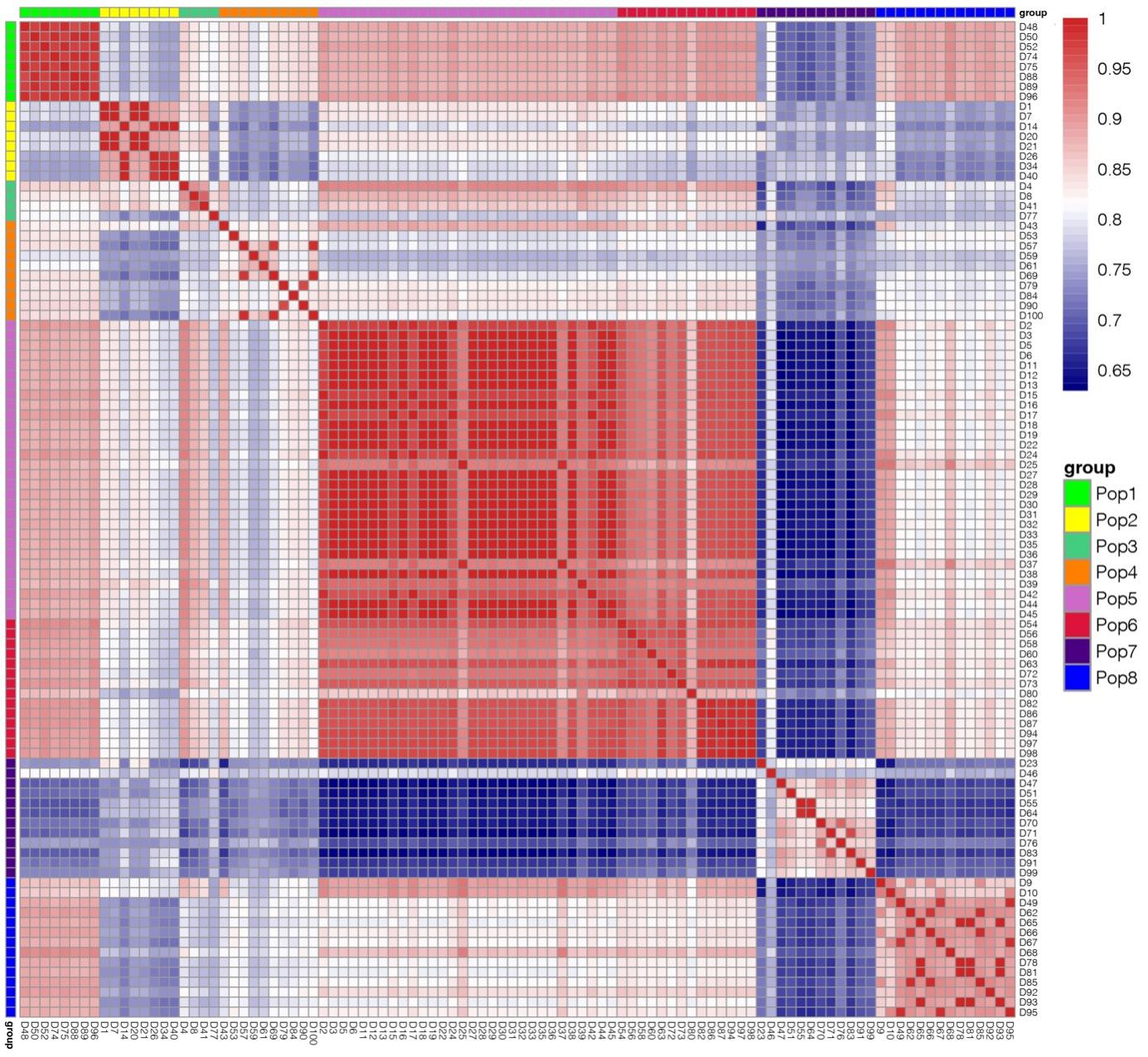


**Supplementary Figure 2.** The heatmap of the phylogenetic relationship of 100 *E. coli* strains. This was drawn using the heatmap package in R language.


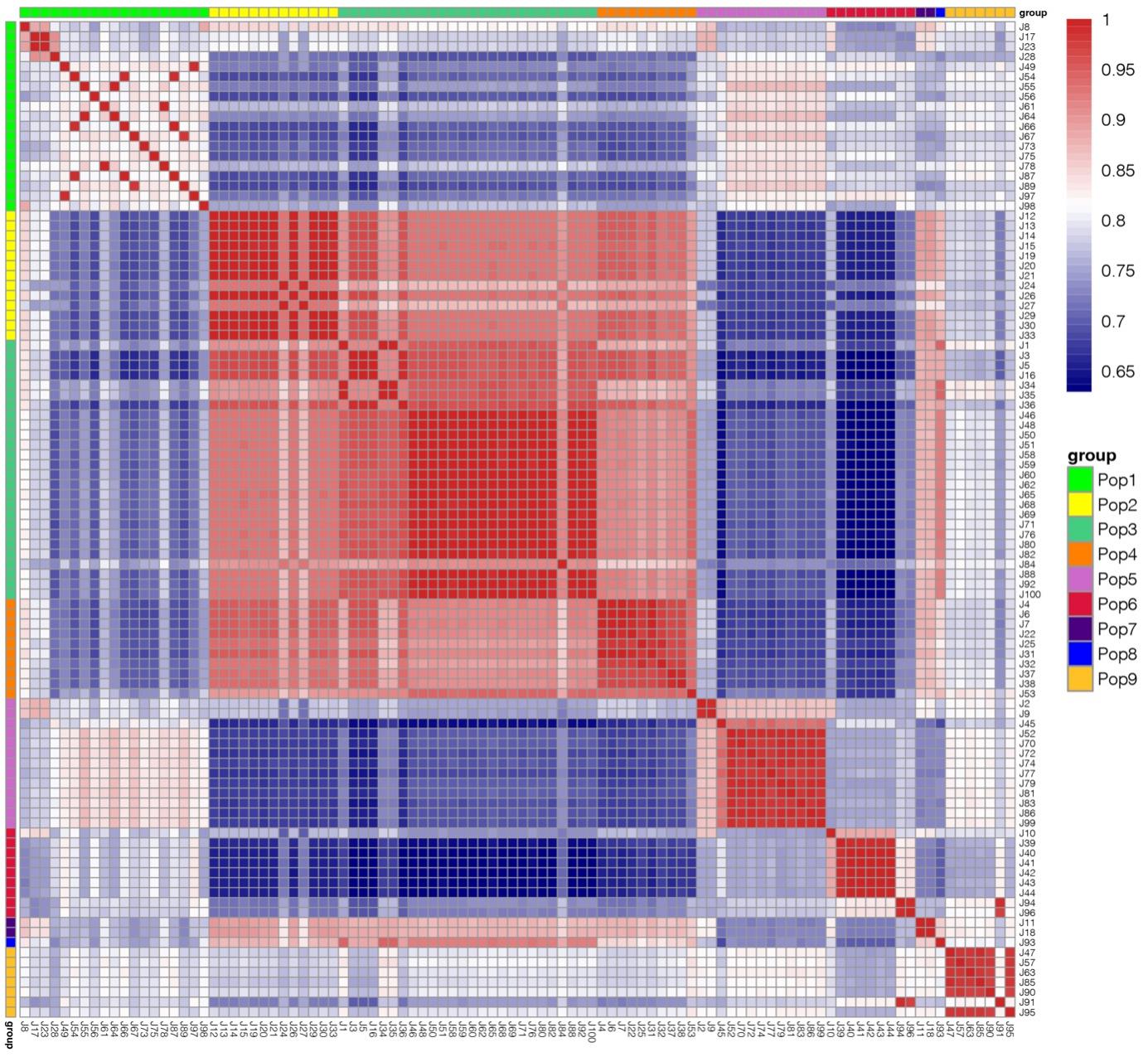


**Supplementary Figure 3.** The heatmap of the phylogenetic relationship of 100 *S. aureus* strains. This was drawn using the heatmap package in R language.


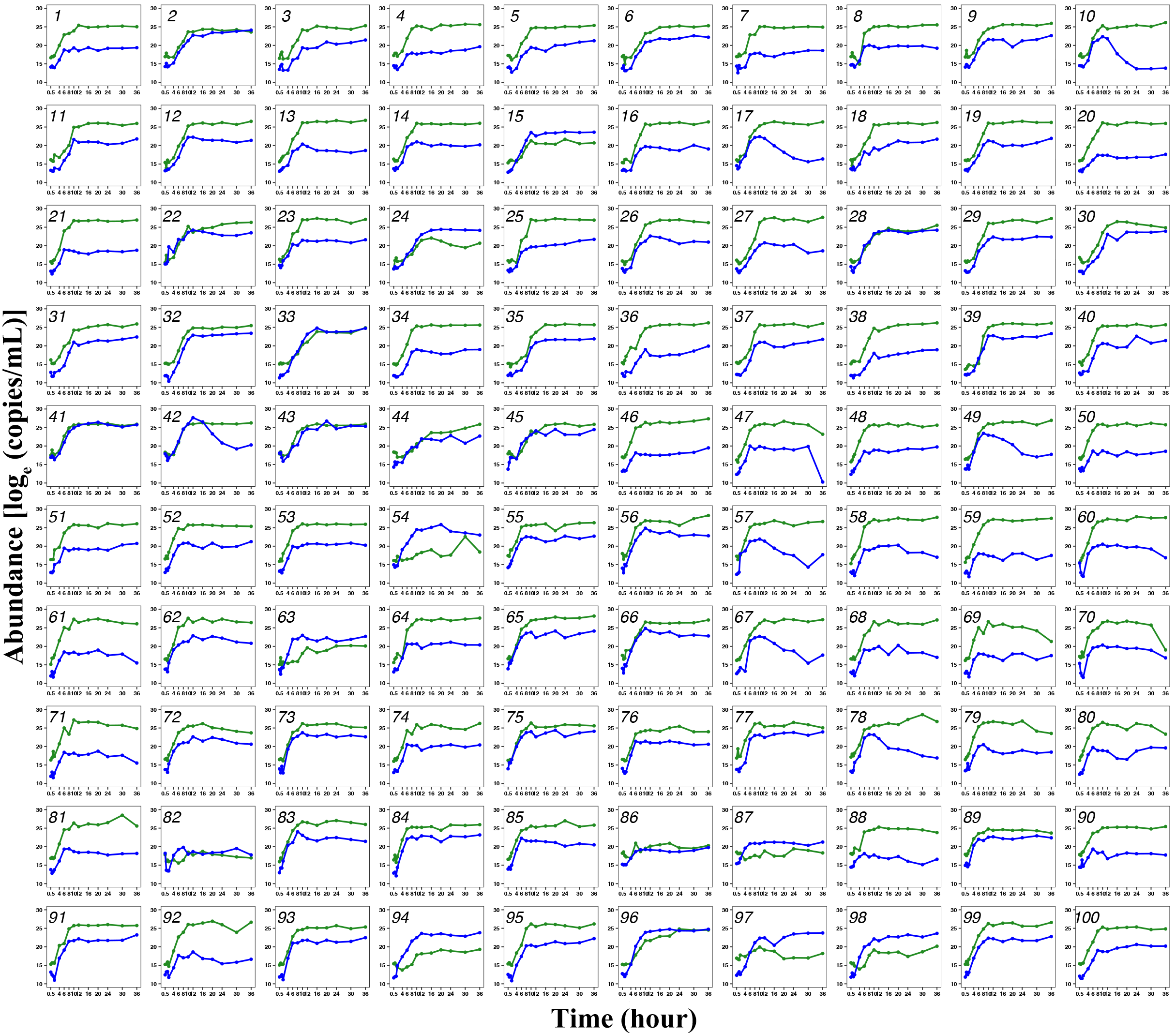


**Supplementary Figure 4.** Actual individual growth curves of 100 pairs of *E. coli* and *S. aureus*. The green line represents *E. coli*, and the blue line represents *S. aureus*. The horizontal axis represents the cultivation time (hour), and the vertical axis represents the logarithm value of microbial abundance.


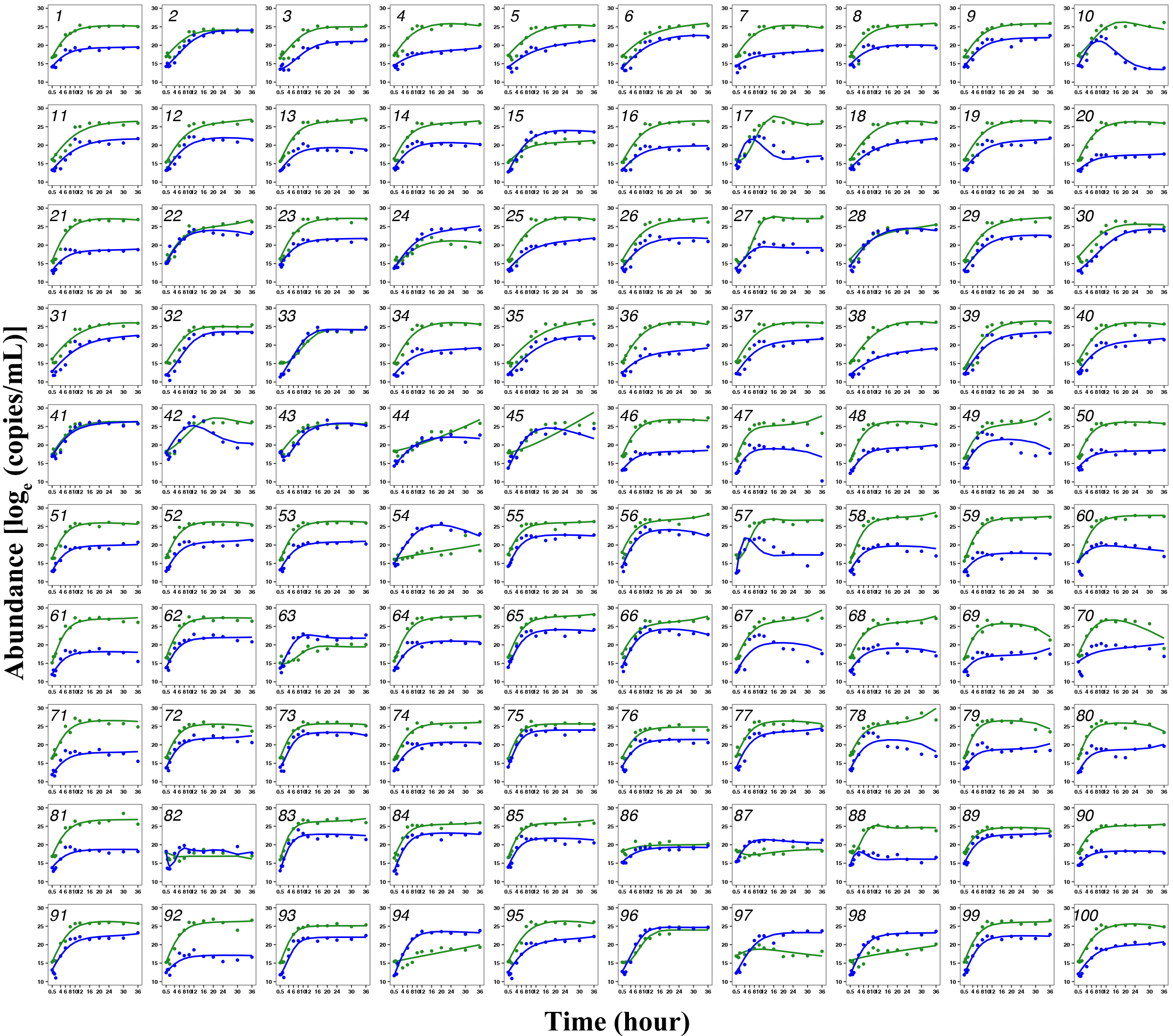


**Supplementary Figure 5.** Fitted individual growth curves of 100 pairs of *E. coli* and *S. aureus*. The green line represents *E. coli*, and the blue line represents *S. aureus*. The horizontal axis represents the cultivation time (hour), and the vertical axis represents the logarithm value of microbial abundance. These phenotypic data were fitted by a set of generalized LV ordinary differential equations.





**Supplementary Figure 6.** The strategy matrix of interaction between *E. coli* and *S. aureus*.





**Supplementary Figure 7.** Frequency of 57 genes from *E. coli* **(A)** and 43 genes from *S. aureus* **(B)** in 244 SNP combinations. The green color represents *E. coli*, and the blue color represents *S. aureus*.
